# Prestimulus oscillatory brain activity interacts with evoked recurrent processing to facilitate conscious visual perception

**DOI:** 10.1038/s41598-022-25720-2

**Published:** 2022-12-22

**Authors:** Kristina Krasich, Claire Simmons, Kevin O’Neill, Charles M. Giattino, Felipe De Brigard, Walter Sinnott-Armstrong, Liad Mudrik, Marty G. Woldorff

**Affiliations:** 1 Center for Cognitive Neuroscience, Duke Institute for Brain Sciences, Duke University, Durham, NC 27708 USA; 2 Department of Psychology and Neuroscience, Duke University, Durham, NC 27708 USA; 3 Our World in Data, Oxford Martin Programme on Global Development, University of Oxford, Oxford, OX1 3BD UK; 4 Department of Philosophy, Duke University, Durham, NC 27708 USA; 5 School of Psychological Sciences, Tel Aviv University, 69978 Tel Aviv, Israel; 6 Sagol School of Neuroscience, Tel Aviv University, 69978 Tel Aviv, Israel; 7 Department of Psychiatry, Duke University, Durham, NC 27708 USA

**Keywords:** Consciousness, Perception

## Abstract

We investigated whether prestimulus alpha-band oscillatory activity and stimulus-elicited recurrent processing interact to facilitate conscious visual perception. Participants tried to perceive a visual stimulus that was perceptually masked through object substitution masking (OSM). We showed that attenuated prestimulus alpha power was associated with greater negative-polarity stimulus-evoked ERP activity that resembled the visual awareness negativity (VAN), previously argued to reflect recurrent processing related to conscious perception. This effect, however, was not associated with better perception. Instead, when prestimulus alpha power was elevated, a preferred prestimulus alpha phase was associated with a greater VAN-like negativity, which was then associated with better cue perception. Cue perception was worse when prestimulus alpha power was elevated but the stimulus occurred at a nonoptimal prestimulus alpha phase and the VAN-like negativity was low. Our findings suggest that prestimulus alpha activity at a specific phase enables temporally selective recurrent processing that facilitates conscious perception in OSM.

## Introduction

*Conscious perception* entails subjectively experiencing external inputs in an accessible and reportable way. Although ubiquitous in our everyday experiences, conscious perception of visual stimuli does not always occur. This has led scientists, clinicians, and philosophers alike to debate what complex, interactive, and covarying mechanisms actually engender conscious visual perception^1,2,4^. In visual processing, there is an initial feedforward sweep of neuronal activity through lower-level visual areas toward higher-order ones^5^. After this feedforward sweep reaches a given visual area, activation propagates back to the lower-level visual areas via feedback connections (i.e., *recurrent processing*)^6,7^. Such feedback connections have been suggested to transmit predictions regarding incoming sensory signals, whereas feedforward connections may transmit residual errors in these predictions that are then used to update the feedforward activation^8^. At least two prominent theories argue that stimulus-elicited recurrent processing at more local-level^9,10,12^ or more global-level interactions (e.g., involving parietofrontal areas)^13–15^ is necessary for conscious perception. Indeed, past research has shown that conscious perception is disrupted when stimulus-elicited recurrent processing is impaired^12,16–20^.

In addition to recurrent processing, however, the neurocognitive state of the brain prior to stimulus presentation may also play an important role in conscious perception. Past research has shown that detection accuracy for difficult-to-detect stimuli was better when prestimulus oscillatory brain activity in the alpha-band (8–12 Hz) was attenuated over posterior cortical brain regions^21–30^, although this effect has not always been observed in stimulus discrimination-oriented tasks^31–34^. Stimulus detection accuracy has also been associated with the stimulus having occurred at a preferred prestimulus alpha phase^22,35–37^, particularly when prestimulus alpha power was elevated^27,28,38^.

In general, alpha is thought to reflect inhibitory mechanisms that vary between states of cortical inhibition and excitation. Alpha power across multiple oscillatory periods inversely scales with cortical excitability, and the phase of the oscillatory period reflects rhythmic fluctuations between maximal excitability and maximal inhibition^39–43^. When prestimulus alpha power is relatively low, sensory neurons with higher levels of excitation are thought to fire tonically and desynchronized from the oscillation, which generally enhances cortical excitability^40^. When prestimulus alpha power is elevated, however, sensory neurons with higher levels of excitation are thought to fire rhythmically and synchronized with the phase of the oscillation, such that a given phase of this oscillation is more inhibitory to those neurons^40^. Some have argued that alpha power somehow impacts conscious perception through a general boost in cortical excitability (for two different views, see^44,45^), whereas alpha phase may facilitate more temporally precise conscious perception (reviewed in^46^).

What remains unclear, though, is whether and how prestimulus alpha activity interacts with stimulus-elicited recurrent processing within the neural cascade that supports conscious perception. One previous study showed that individuals with higher peak prestimulus alpha frequencies (i.e., shorter alpha periods) showed earlier stimulus-elicited peak latencies of the P2 event-related potential (ERP) component, and thus the authors suggested that prestimulus alpha might influence stimulus-elicited recurrent processing^47^. There are a few ways prestimulus alpha power and phase might impact recurrent processing on the way to conscious perception. When prestimulus alpha power is attenuated, it is possible that the neural connections necessary for recurrent processing are generally innervated, thus facilitating subsequent stimulus-elicited recurrent processing. Here, phase would have little to no impact given the tonic, desynchronized firing of these neural connections^40^. When prestimulus alpha power is elevated, though, these neural connections would not be generally innervated, but if the stimulus occurred at an optimal, non-inhibitory phase of the alpha oscillation, it might enable temporally selective stimulus-elicited recurrent processing. This temporal selectivity could be a mechanism by which alpha phase enables temporally precise conscious perception for rapidly occurring visual stimuli^46^. As such, the current work examined the possible link between prestimulus alpha activity (power and phase) and stimulus-elicited recurrent processing on the way toward conscious perception, a neurocognitive relationship that has heretofore been little investigated.

The current work made use of a spatial-cueing study that had examined ERP activity related to conscious perception^48^. Specifically, in this task, conscious perception was manipulated using object substitution masking (OSM), a masking technique thought to disrupt recurrent processing while preserving the initial feedforward signal^16,17,49–53^. In a typical OSM paradigm, a brief stimulus and a surrounding, nonspatially-overlapping, four-dot mask onset simultaneously. If the stimulus and mask offset simultaneously, the stimulus is likely to be consciously perceived. However, when the mask lingers after stimulus-offset, the stimulus is substantially less likely to be consciously perceived^51^. It has been argued that the lingering mask evokes additional feedforward processing of the mask-only representation that disrupts recurrent processing of the stimulus + mask representation, either replacing^49–52^ or updating^53–55^ the latter with the former.

**Figure 1:**
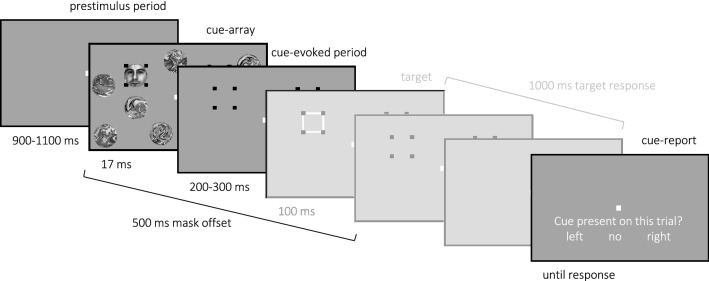
A sample trial sequence. After a jittered prestimulus fixation period, a transient cue-array appeared, which contained distractors and two potential cue-locations that were each denoted with a four-dot OSM mask. On 80% of trials, a cue appeared within one of the cue-locations. In 70% of trials, a target then appeared at one of the cue-locations after a jittered ISI (note that the target sequences and responses were not investigated in the current work and are thus greyed out in this illustration). On every trial, the mask-offset and the time allocated for target response was fixed. At the end of each trial, participants reported the location of the cue or indicated that the cue was absent. *Cue-perceived* trials were operationalized as trials when the participant correctly discriminated the location of the cue, and *not-perceived* trials were operationalized as trials when the participants incorrectly indicated that the cue was absent. This figure was adapted from Figure 1 of Giattino et al.^48^ and is not drawn to scale.

In the Giattino et al.^48^ spatial-cueing paradigm, the masked stimulus was a lateralized cue (a face or house stimulus) that, in 80% of trials, could appear in one of two cue-locations that were each denoted by a four-dot OSM mask (Fig. 1). The masked locations were always symmetric across the vertical midline and randomly appeared in either the upper or lower visual fields. When the cue would offset, the four-dot masks remained on the screen for 500 ms to elicit the typical OSM effect (previously demonstrated in Giattino et al.,^48^). The cue was absent in 20% of trials, but the masked locations would still appear. At the end of each trial, participants were prompted to report the cue’s location (left or right) or its absence. *Cue-perceived* trials were operationalized as trials in which participants correctly discriminated the location of a present cue, and *not-perceived* trials were those that participants incorrectly reported that a present cue was absent.

Findings from Giattino et al.^48^ showed no difference in the cue-evoked P1 ERP component for the cue-perceived versus the not-perceived trials, evidencing that the OSM sequence did not disrupt feedforward processing. Instead, there tended to be a greater posterior negativity from 150 to 250 ms for cue-perceived compared to not-perceived trials, a negative-wave difference that resembled the *visual awareness negativity* (VAN)^1,56,57^. Past research has shown that the VAN is a negative-polarity posterior difference between ERPs evoked by stimuli reported as perceived versus not-perceived stimuli, occurring at about 200 ms post-stimulus-onset^58–62^. This activity is thought to reflect local recurrent processing along the visual hierarchy^1,56,57^, and thus Giattino et al.^48^ concluded that the observed VAN reflected the relative disruption of recurrent processing on trials when the cue was not consciously perceived.

In the current work, we first compared prestimulus alpha power and phase between cue-perceived and not-perceived trials recorded in Giattino et al.^48^. These analyses provided initial insights into whether prestimulus alpha activity was related to cue perception in OSM. For instance, although past findings have been somewhat mixed, it was possible that prestimulus alpha power would be lower for cue-perceived than not-perceived trials^21–30^. We also predicted an effect of phase, given the need in OSM for temporally precise visual perception to distinguish the brief cue + mask stimulus from the lingering mask-only stimulus. Specifically, we predicted that cue-perceived and not-perceived trials might be associated with opposite preferred prestimulus alpha phase^22,35–37^. In addition, if there was also an effect of power, it was possible that the phase effect would only be observed when prestimulus alpha power was high and not when it was low^27,28,38^.

We then considered whether the relationship between prestimulus alpha and cue perception was at least in part due to how prestimulus alpha might impact stimulus-elicited recurrent processing. Specifically, we looked at how prestimulus alpha impacted modulations in the cue-evoked ERP activity that was temporally consistent with the VAN effect observed 150–250 ms post cue-onset in Giattino et al.^48^—termed hereafter as the *VAN-window ERP*. If prestimulus alpha power facilitates conscious perception in OSM by innervating the neural connections necessary for stimulus-elicited recurrent processing, we predicted that trials with lower prestimulus alpha power should correspond with a greater VAN-window ERP negativity—and then more accurate cue-perception—relative to trials with higher prestimulus alpha power. If prestimulus alpha phase facilitates conscious perception in OSM through temporally selective stimulus-elicited recurrent processing, we predicted that more negative and less negative VAN-window ERP responses would be associated with opposite preferred prestimulus alpha phases. Again, especially if there was an effect of power, it was also possible that the effect of phase on VAN-window ERP activity would be primarily observed on trials when prestimulus alpha power was high. These collective findings would thus delineate key parts of the cascading neurocognitive pathway that leads from levels of cortical excitation/inhibition to evoked recurrent processing and on to eventual conscious perception.

## Results

### Reports of cue-perception

Data from twenty-nine right-handed participants (*M* age = 19.28, *SD* age = 2.22, female = 15) from Giattino et al.^48^ were analyzed for the current report. Cue-present trials consisted of 80% of all trials. In these cue-present trials, participants on average correctly reported the location of the cue (cue-perceived) on 43% (*SE* = 3%) of trials and incorrectly reported that the cue was absent (not-perceived) on 50% (*SE* = 3%) of trials. Only in 7% (*SE* = 1%) of the cue-present trials did they report perceiving that the cue was present but incorrectly reported its location. These trials were excluded from the current work because it was unclear whether participants truly perceived the cues on these trials. Additionally, a trial-level Bayesian generalized mixed-effect regression analysis that modeled *cue-perception* (0 or 1) with *cue type* (house [reference] or face) as a fixed effect and with random intercepts and slopes for cue type for each participant showed no evidence that participants discriminated cue-location differently for face-cues than house-cues (*b* = 0.06, 95% HDI = [− 0.13, 0.25], BF = 0.12). Accordingly, in all our analyses, we collapsed across trials with face-cues and house-cues.

### Examining prestimulus alpha activity and reported cue perception

**Figure 2:**
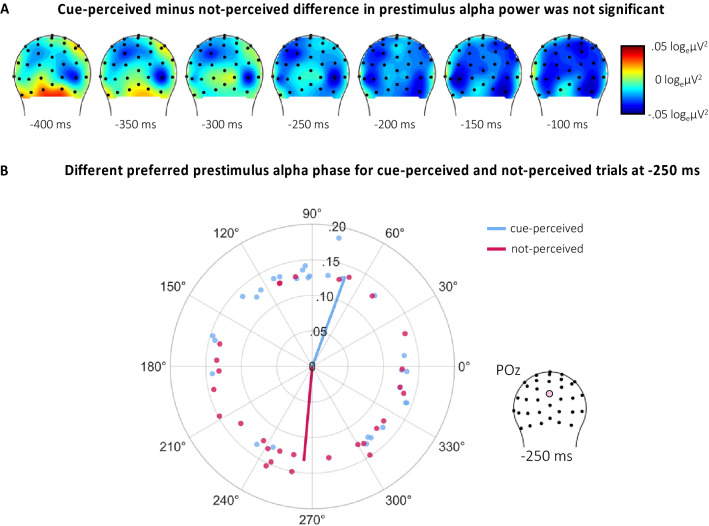
(**A**) The topographic distribution of prestimulus alpha power (8–12 Hz) for cue-perceived minus not-perceived trials. To test this difference, power was averaged at -250 ms pre-cue across 8–12 Hz and across an occipitoparietal region of interest (ROI). However, a Bayesian generalized mixed-effect regression analysis showed no statistical evidence that prestimulus alpha power differed between cue-perceived and not-perceived trials. (**B**) The average preferred prestimulus alpha phase for cue-perceived (blue lines) and not-perceived trials (red lines). A Bayesian generalized mixed-effect regression analysis showed moderate evidence that these preferred prestimulus alpha phases were different between these conditions. The vector length represents intertrial phase coherency (ITPC). Data points reflect the preferred phase and ITPC for individual subjects. Phase measures of the prestimulus alpha activity were extracted from a window centered at -250 ms (relative to cue stimulus onset) at electrode site POz.

Prestimulus alpha power and phase were first compared between cue-perceived and not-perceived trials from Giattino et al.^48^ to provide initial insights into whether prestimulus alpha activity was related to cue perception in OSM. Prestimulus alpha power at -250 ms pre-cue was averaged across 8–12 Hz and across an occipitoparietal region of interest (ROI) where prestimulus alpha power effects have been observed in past research (see Methods). Illustrated in Fig. 2A, posterior prestimulus alpha power was lower in cue-perceived than not-perceived trials. However, a Bayesian generalized mixed-effect regression, with *perception* (not-perceived [reference level] or cue-perceived) as a fixed-effect and with random intercepts for each *participant*, showed no statistical evidence of this effect (*b* = − 0.02, 95% HDI = [− 0.07, 0.03], BF = 0.04).

We tested for phase effects across cue-perceived and not-perceived trials irrespective of power because there was no observed power effect. A bootstrapping method was used to separately compute the preferred phase for cue-perceived and not-perceived trials measured at -250 ms pre-cue and at electrode site POz. A Bayesian generalized mixed-effect regression was used to model *preferred phase* (in radians) with *perception* (not-perceived [reference level] or cue-perceived) as a fixed-effect and with random intercepts for each *participant*. Illustrated in Fig. 2B, the findings showed moderate evidence that the preferred phase for cue-perceived trials (*M* = 70°, *SD* = 70°) was different than the preferred phase for not-perceived trials (*M* = 265°, *SD* = 69°) (*b* = 2.31, 95% HDI = [− 3.14, − 1.79] [− 0.25, 3.14], BF = 6.14).

### Examining how the VAN-window ERP varied with prestimulus alpha power

We next examined whether prestimulus alpha power was related to cue-evoked VAN-window ERP activity. To provide initial insights, we organized trials into terciles according to ascending prestimulus alpha power as averaged within the aforementioned ROI. Thus, we created three categorical conditions, with the *low-power* and *high-power* conditions consisting of the terciles with the lowest and highest averaged prestimulus alpha power respectively. ERPs were then selectively averaged across the trials within each alpha power condition time-locked to the onset of the cue.

**Figure 3:**
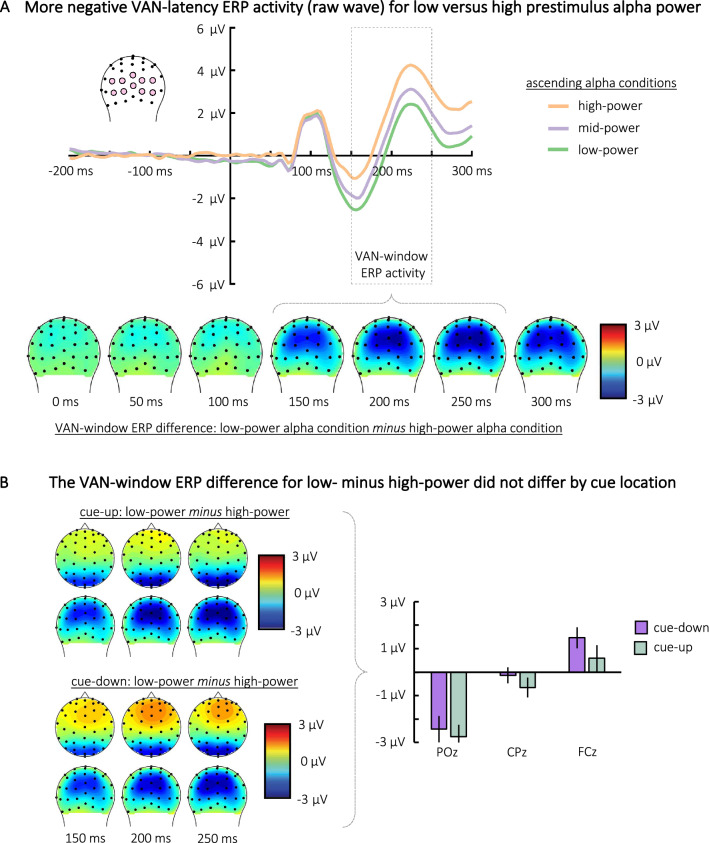
(**A**) VAN-window ERP activity for trials with low, mid, and high prestimulus alpha power. Alpha conditions were created by organizing trials into terciles according to ascending prestimulus alpha power as averaged within a specific spatiotemporal ROI. Cue-locked ERP raw traces for each prestimulus alpha condition are plotted (top panel) along with the topographic distribution for the difference wave derived from the low minus high alpha conditions (bottom panel). A Bayesian mixed-effect regression showed strong evidence that VAN-window ERP amplitudes were more negative for the low-power versus the high-power alpha conditions. This indicates that lower prestimulus alpha was associated with more negative VAN-window ERP amplitudes. (**B**) The left-side topo plots illustrate the ‘low-power minus high-power’ VAN-window ERP difference for trials when the cue appeared in the upper visual field (cue-up) and for trials when the cue appeared in the lower visual field (cue-down). Illustrated in the right-side bar plot, a Bayesian generalized mixed-effect regression showed no difference in amplitude between the ‘cue-up’ and ‘cue-down’ trials across three medial electrode sites.

Illustrated in Fig. 3A, there was a posterior negative polarity difference observed between the low-power and high-power conditions that was temporally consistent with the VAN. To test this effect, VAN-window ERP amplitudes for low-power and high-power conditions were averaged across 150–250 ms and across the same posterior ROI that was used for creating the prestimulus alpha conditions. To simplify our statistical approach, we only compared averaged ERP amplitudes between the low-power and high-power conditions. *VAN-window ERP amplitudes* were modeled with a Bayesian generalized mixed-effect regression analysis with *alpha condition* (high-power [reference level] or low-power) as a fixed-effect and with random intercepts for each *participant*.

The findings showed strong evidence that the VAN-window ERP amplitudes were more negative for the low-power condition compared to the high-power condition (*b* = − 1.39, 95% HDI = [− 2.11, − 0.63], BF = 113.06). This indicates that lower prestimulus alpha power was associated with a more negative VAN-window ERP elicited by the cue stimulus. However, a Bayesian repeated measures ANOVA showed no evidence that the rate of cue-perceived trials differed across the low-power (*M* = 43%, *SE* = 3%) and high-power (*M* = 43%, *SE* = 3%) conditions, BF = 0.30. This indicates that the relationship between lower prestimulus alpha power and a greater VAN-window ERP negativity did not correspond with better cue-perception.

#### Topographic distribution of the VAN-window ERP effect

We next investigated the topographic distribution of this VAN-window ERP negativity difference, which could provide suggestive insights into the possible cortical source of the VAN-window ERP activity. This analysis was motivated by several converging literatures. First, recurrent processing to the low-level V1/V2 areas within the visual cortex has been shown to facilitate conscious visual perception^10–12^, affording the possibility that VAN-window ERP activity reflects recurrent processing to these lower-level visual areas. In contrast, studies investigating the source of the scalp-recorded VAN have suggested that it originated along the ventral visual stream in an occipital-temporal area beyond V1/V2^61,62^. Possible still, the spatial task-specifics of our paradigm may have encouraged activation within the dorsal visual pathway, which has been shown to be important in visuospatial perception (reviewed in^63,64^). It was thus possible that the VAN-window ERP observed in the current work was generated during recurrent processing within the dorsal visual pathway.

To assess these possibilities, we used a previously established approach for estimating the possible source of early-latency visually-evoked ERPs^65,66^. ERPs are thought to originate from the dendritic trees of large pyramidal neurons that are aligned orthogonally to the cortical surface^67^. This results in dipoles that are oriented perpendicular to the cortical surface and vary according to the cortical folding of a given region^67^.

Following the neuroanatomic folding of the early visual cortex, inputs from the upper visual field are generally represented along the ventral surface of the downward-facing striate cortex in the calcarine fissure (i.e., V1) along with portions of the extrastriate cortex near it (i.e., V2), whereas inputs from the lower field are represented along the dorsal portions of these areas. Thus, visually evoked potentials originating from these cortical regions tend to show a topographic polarity inversion posteriorly as a function of whether the stimulus appeared within the upper versus lower visual field^66,68–72^. Accordingly, in the current work, if the observed VAN-window ERP difference between the low-power and high-power conditions represented recurrent activity originating largely from these low-level visual cortical areas, there should be a posterior polarity inversion in this effect for trials with cues appearing in the lower verses upper visual field. In contrast, if this effect originated beyond these low-level visual cortical areas, there should not be a posterior visual-field polarity inversion for the ERP difference across alpha conditions.

We leveraged the fact that the cues in the present study were randomly presented from in the lower visual field (50% of trials) or upper visual field (50% of trials). We computed the average difference in the VAN-window ERP between the low- and high-power prestimulus alpha conditions from 150 to 250 ms post-cue separately for trials when the cue appeared in the lower visual field (cue-down) and those when it appeared in the upper visual field (cue-up). Then, to examine the distribution differences across the head as a function of upper verses lower field, we extracted and compared this average difference from three medial electrode sites (POz, CPz, FCz) for cue-up and cue-down trials.

Illustrated in Fig. 3B, both for cue-down and cue-up trials, the topography of the VAN-window negative polarity difference between low- and high-power conditions showed a posterior negativity that progressed into a smaller anterior positivity. This topography was modeled with a Bayesian generalized mixed-effect regression as a *cue location* (cue-down [reference level] or cue-up) by *electrode* (POz [reference level], CPz, FCz) interaction with random intercepts for each *participant*. The findings showed no effect of cue location (*b* = − 0.64, 95% HDI = [− 1.55, 0.19], BF = 1.41). There was an effect of electrode, with CPz (*M* = − 0.39 µ, *SE* = 0.34 µ) showing a less negative amplitude difference compared to POz (*M* = − 2.59 µ, *SE* = 0.52 µ) (*b* = 1.45, 95% HDI = [0.51, 2.39], BF = 46.61). Further, FCz (*M* = 1.0 µ 3, *SE* = 0.46 µ) showed a positive amplitude difference (i.e., low-power condition amplitude was *greater* that the high-power condition) that was greater than POz (*b* = 2.78, 95% HDI = [1.83, 3.71], BF = 3.56e+5). There was no cue location by CPz interaction (*b* = 0.36, 95% HDI = [− 0.86, 1.50], BF = 0.70), and no cue location by FCz interaction (*b* = 0.29, 95% HDI = [− 0.92, 1.45], BF = 0.66).

These findings thus provide two suggestive insights. First, there was no evidence of a posterior visual-field polarity inversion in the ERP difference for cue-up and cue-down trials, an effect that would have been consistent with a cortical source originating from low-level visual cortical areas (i.e., V1 and V2). There was, however, a posterior-to-anterior inversion, regardless of cue-location. Considering this finding along with the spatial-task specifics of our paradigm, we speculate that the dorsal parietal cortex was a plausible source of the posterior VAN-window ERP negativity effect and that the corresponding broadly distributed, anterior positivity could reflect a polarity inversion from this posterior negativity (i.e., reflected the other side of the dipole). However, scalp topography provides rather limited insights into cortical sources (e.g.,^73^), and thus future work would be needed to identify the exact source of the VAN-window ERP activity in OSM.

### Prestimulus alpha activity on VAN-window ERP toward conscious perception

**Figure 4:**
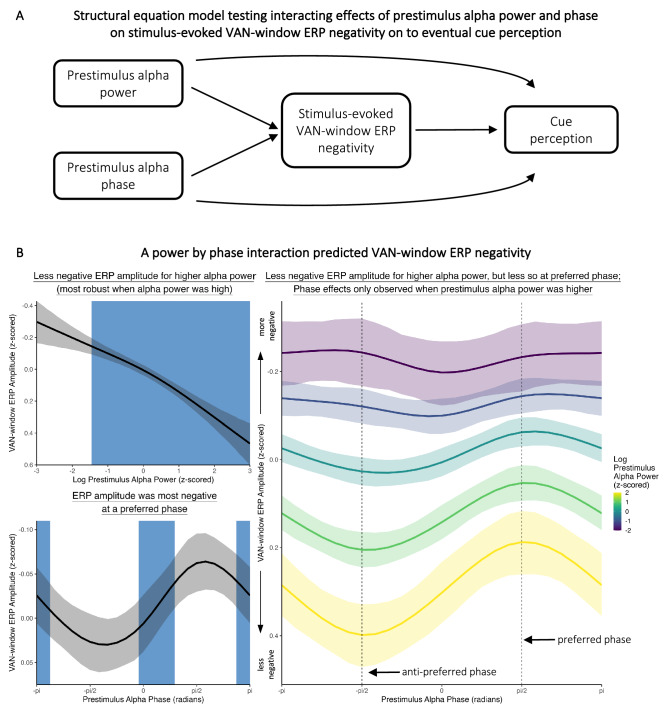
(**A**) Structural equation model with non-linear smooth terms testing (1) the effects of prestimulus alpha power and phase on VAN-window ERP amplitude and (2) the effects of prestimulus alpha power and phase and VAN-window ERP amplitude on cue perception. (**B**) The effects of prestimulus alpha power and phase on VAN-window ERP amplitude. The left panels show the simple effects of prestimulus alpha power (top) and phase (bottom). The blue shaded regions illustrate slopes where BF > 10. Prestimulus alpha power predicted VAN-window ERP amplitude, such that higher alpha power was associated with less negative VAN amplitudes (i.e., a smaller VAN). This effect was primarily observed when prestimulus alpha power was above the mean. Prestimulus alpha phase also predicted VAN-window ERP amplitude, such that more negative amplitudes were associated with a preferred phase, and less negative amplitudes were associated with an anti-preferred phase. These effects were characterized by an alpha power by alpha phase interaction, where the effects of phase were mostly observed when prestimulus alpha power was high.

The findings thus far provided suggestive insights into the relationship between prestimulus alpha activity (power and phase), stimulus-elicited VAN-window ERP activity, and cue perception. To formally test these relationships, we used a trial-level Bayesian structural equation model with non-linear smooth terms to concurrently investigate (1) the effects of prestimulus alpha power and phase on stimulus-evoked VAN-window ERP amplitudes, and (2) the effects of prestimulus alpha activity (power and phase) and VAN-window ERP amplitude on cue perception. The specific causal structure of this model is illustrated in Fig. 4A.

To conduct this analysis, for each trial, we calculated a single measure of prestimulus alpha power, prestimulus alpha phase, and VAN-window ERP amplitude for each trial. Specifically, prestimulus alpha power was averaged at -250 ms across 8–12 Hz and across the same occipitoparietal spatial ROI that was used to create the tercile conditions. Prestimulus alpha phase was again measured at -250 ms at electrode POz. VAN-window ERP amplitude was averaged across 150–250 ms post stimulus onset and across the same spatial ROI as prestimulus alpha power. Prestimulus alpha power and VAN-window ERP amplitudes were then *z*-scored standardized for each participant, so that participant-level means were zero-centered with a standard deviation of 1.

We then modeled trial-level *VAN-window ERP amplitude* as a *prestimulus alpha power* by *prestimulus alpha phase* two-way non-linear interaction. Cue perception (not-perceived or perceived) was modeled as a *prestimulus alpha power* by *prestimulus alpha phase* by *VAN-window ERP amplitude* three-way non-linear interaction with random intercepts for each *participant*.

As illustrated in Fig. 4B, prestimulus alpha power predicted VAN-window ERP amplitudes: higher prestimulus alpha power was associated with less negative VAN-window ERP amplitudes (i.e., a less negative VAN) (*b* = 0.16, 95% HDI = [0.08, 0.25], BF = 20.10). Prestimulus alpha phase also predicted VAN-window ERP amplitudes: more negative amplitudes (i.e., a more negative VAN) were associated with a preferred phase (*b* = − 0.05, 95% HDI = [− 0.09, − 0.02], BF = 44.78), and less negative amplitudes were associated with an opposite, anti-preferred phase (*b* = 0.04, 95% HDI = [0.02, 0.06], BF = 880.48). Phase effects were mostly observed, however, when prestimulus alpha power was at or above its mean, as there was strong evidence for a power by phase interaction (*b* = − 0.03, 95% HDI = [− 0.06, − 0.01], BF = 12.77). This interaction indicated that even when prestimulus alpha power was elevated, a relatively more negative VAN-window ERP amplitude could still be observed at a preferred (versus the anti-preferred) prestimulus alpha phase.

**Figure 5:**
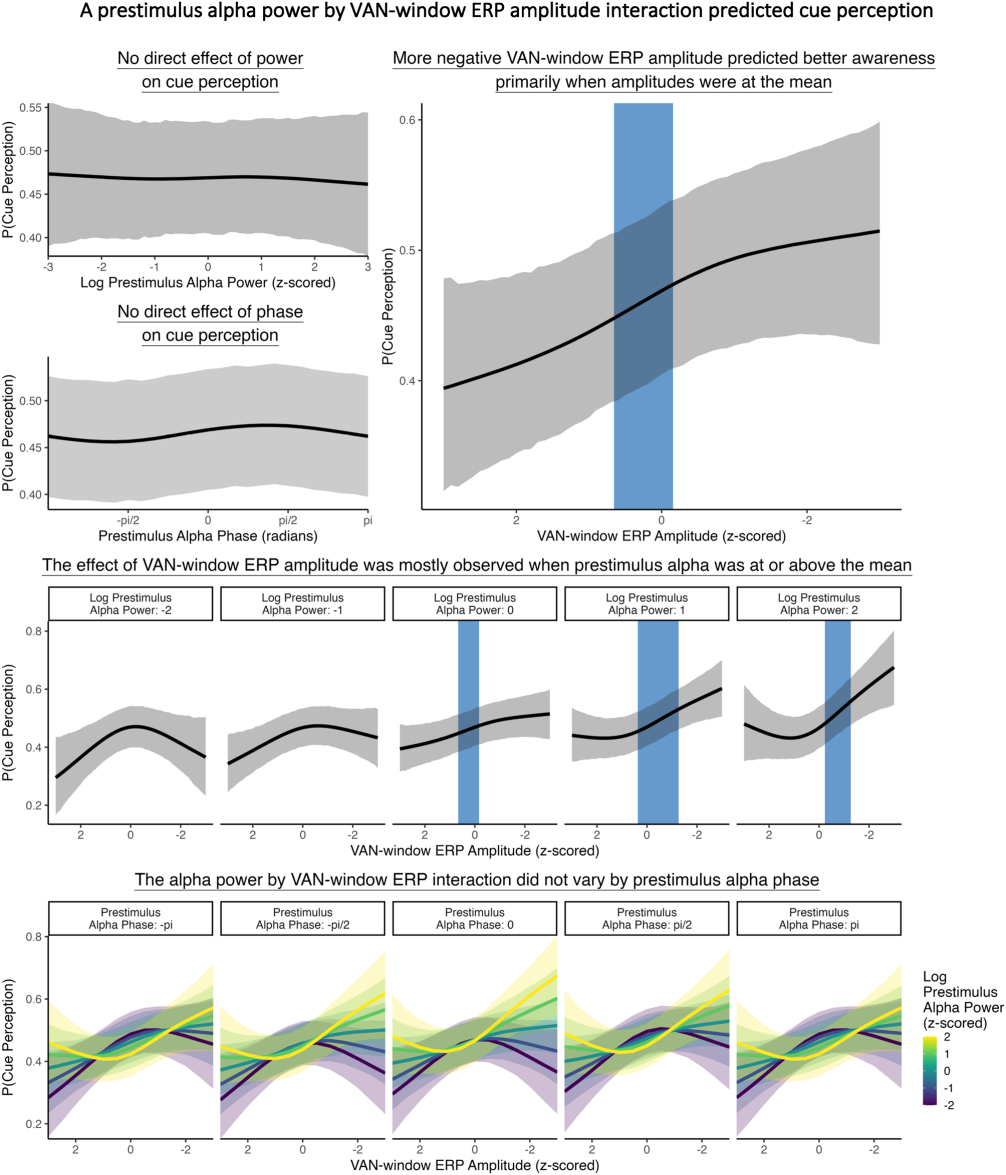
The effects of prestimulus alpha (power and phase) and VAN-window ERP amplitudes on cue perception. A more negative VAN-window ERP amplitude was associated with better cue perception (upper right panel). This effect was mostly observed when prestimulus alpha power was elevated, but there was only anecdotal evidence of this interaction (middle panel). There also was no three-way interaction (bottom panel). Blue shaded regions represent where slopes were BF > 10 for the simple effects and the prestimulus alpha power by VAN-window ERP amplitude interaction.

Illustrated in Fig. 5 (top right panel), better cue perception was associated with more negative VAN-window ERP amplitudes but mostly when VAN-window ERP amplitudes were near the mean (*b* = − 0.08, 95% HDI = [− 0.13, − 0.04], BF = 41.71). This VAN-window ERP effect on cue perception was mostly observed when prestimulus alpha power was elevated (Fig. 5 middle panel), although there was only anecdotal evidence for this prestimulus alpha power by VAN-window ERP amplitude interaction (*b* = − 0.08, 95% HDI = [− 0.13, − 0.03], BF = 3.88). The VAN-window ERP effect on cue perception was similar across different prestimulus alpha phases, as there was no VAN-window ERP by prestimulus alpha phase interaction (*b* = 0.04, 95% HDI = [− 0.02, 0.10], BF = 0.99). Furthermore, there was no evidence of a three-way interaction (Fig. 5 bottom panel) (*b* = − 0.02, 95% HDI = [− 0.05, 0.01], BF = 1.39). There was also no simple effect of prestimulus alpha power on cue perception (*b* = − 0.01, 95% HDI = [− 0.08, 0.05], BF = 0.03), and no simple effect of prestimulus alpha phase at the preferred (*b* = − 0.02, 95% HDI = [− 0.05, 0.01], BF = 0.99) or the anti-preferred (*b* = 0.02, 95% HDI = [− 0.01, 0.06], BF = 1.02) phases (Fig. 5 top left panels).

Considered together, our findings from the trial-level Bayesian structural equation model showed that (1) when prestimulus alpha power was elevated, a preferred prestimulus alpha phase was associated with a more negative VAN-window ERP, and (2) this more negative VAN-window ERP corresponded with better cue perception. Although lower prestimulus alpha power also corresponded with a more negative VAN-window ERP, this effect did not result in better cue perception. Altogether, these results indicated that the mechanism by which prestimulus alpha power and phase impacted cue perception in this OSM task was through their respective effects on the VAN-window ERP.

## General discussion

The current work investigated how the possible relationship between prestimulus alpha activity and stimulus-elicited recurrent processing may cascade into later conscious visual perception. Prestimulus oscillatory and stimulus-evoked ERP data were analyzed from participants who completed a spatial-cuing paradigm where they had to accurately discriminate the left or right location of a brief cue that was perceptually masked through OSM. In OSM, recurrent processing for the rapidly presented stimulus + mask stimulus is thought to be disrupted by the subsequent feedforward processing of the lingering mask-only stimulus. In the current work, we investigated how prestimulus alpha power and phase might facilitate recurrent processing for the cue + mask representation toward accurate conscious perception of the cue in the presence of OSM.

We found no overall difference in prestimulus alpha power between cue-perceived and not-perceived trials. Although this finding is *inconsistent* with some past studies that have observed this difference^21–30^, it is *consistent* with other stimulus discrimination-oriented tasks that have not^31–34^. Lower prestimulus alpha power was, however, associated with a substantially greater negative-polarity cue-evoked posterior VAN-window ERP wave. The topographic distribution of this effect—a posterior negativity to an anterior positivity—offered several suggestive insights. First, although scalp topography provides rather limited insights into cortical sources^73^, the topographic distribution was consistent with a contributing source somewhere within the dorsal parietal cortex. This aligns with past research implicating a critical role of the dorsal visual pathway in visuospatial perception as well as the visuospatial demands to correctly discriminate the cue location in our task^63,64^. Moreover, the topographic distribution we observed was inconsistent with past work suggesting that the VAN originates somewhere along the ventral visual stream^1,56,57,61,62^. It is possible that the VAN-window negativity is not specific to any one specific neural generator, but rather it can take place at different neural loci in the context of different tasks (akin to the claim that the VAN is a sub-category of the more general Perceptual-Awareness-Negativity that generalizes to other sensory domains^56^). This possibility should be further tested in future studies investigating the neural generators of the VAN.

Given that the VAN has been argued to reflect recurrent processing^1,56,57^, the effect of prestimulus alpha power on the VAN-window ERP negativity suggests that with lower prestimulus alpha power, the neural connections that are necessary for stimulus-elicited recurrent processing were innervated. Critically, though, this effect—without any consideration of prestimulus alpha phase—did *not* correspond with better cue-perception. Thus, simply innervating the neural connections for recurrent processing did not seem sufficient for enabling accurate conscious perception in this task.

Instead, accurate conscious perception in our OSM task was most related to how elevated prestimulus alpha power and specific prestimulus alpha phases interactively impacted subsequent cue-evoked VAN-window ERP activity. Our findings from the trial-level Bayesian structural equation model showed that when prestimulus alpha power was elevated, a preferred prestimulus alpha phase was associated with a more negative VAN-window ERP. This more negative VAN-window ERP corresponded with better cue perception. Conversely, when prestimulus alpha power was elevated, an anti-preferred prestimulus alpha phase was associated with a less negative VAN-window ERP, which was then associated with worse cue perception.

Considering these findings, we propose that in OSM, when the cue + mask stimulus appears at an optimal, excitatory phase of the alpha oscillation, recurrent processing for the cue + mask stimulus will be more resilient to suppression from the feedforward processing of the subsequent mask-only stimulus that stays on the screen after the cue offset. This effect will then manifest as a greater VAN-window ERP negativity (i.e., greater VAN) and contribute to a greater likelihood of conscious visual perception. In contrast, when the cue + mask stimulus onsets at a nonoptimal, inhibitory phase of the oscillation, the elicited recurrent processing will be less resilient and thus more susceptible to being disrupted by the feedforward processing of the lingering mask-only stimulus. This will manifest as a less negative VAN-window ERP response (smaller VAN) and will decrease the likelihood of conscious visual perception. Critically, though, this effect will primarily occur when prestimulus alpha power is *elevated* because the associated sensory neurons with high levels of excitation will fire rhythmically and synchronized with the oscillation^40^. In this way, our findings suggest that prestimulus alpha phase facilitates the *temporally selective* activation of feedforward-feedback interactions. These interactions can either facilitate or impair conscious perception in OSM depending on whether the stimulus appeared at a preferred or anti-preferred alpha phase.

When prestimulus alpha power was low, trials with the most negative VAN-window ERP amplitudes did not show a different preferred prestimulus alpha phase than trials with the least negative ERP response. The rate of cue-perception also did not vary between these conditions. These collective findings further support the idea that during a state of cortical excitability—when sensory neurons with high levels of excitation are firing tonically and desynchronized from the alpha oscillation^40^—conscious perception of rapidly occurring transient stimuli is not modulated by the alpha phase. Thus, we argue that general cortical excitability does not provide the temporally selective activation of feedforward-feedback interactions that is necessary in OSM to protect the cue + mask elicited recurrent processing from the subsequent mask-only feedforward processing.

In conclusion, our findings showed that conscious perception of stimuli during OSM in part depended on the prestimulus neurocognitive state of the brain, including its relationship to the recurrent processing elicited by the stimulus. These findings thus help to delineate key parts of the cascading neurocognitive pathways that lead from cortical excitation/inhibition to evoked recurrent processing and on to eventual conscious perception. As such, the current work provides critical insights about how the prestimulus neurocognitive state of the brain, as indexed by the power and phase of alpha oscillations, is related to the stimulus-elicited recurrent processing that engenders conscious visual perception.

## Methods

### Participants

The 40 participants in the main study of Giattino et al. were recruited from Duke University and the local community. They received course credit or monetary compensation for participation. Informed consent was obtained from each participant in accordance with approval from the Duke University Medical Center Institutional Review Board.

One participant was excluded due to a technical error in the recording of the EEG data, seven participants were excluded for having too few either *cue-perceived* or *not-perceived* trials (< 30 trials). Three additional subjects were removed for being left-handed. Therefore, data from twenty-nine right-handed participants (*M* age = 19.28, *SD* age = 2.22, 15 = female) were analyzed in the current work.

### Stimuli and procedures

All stimuli and study procedures were in accordance with the guidelines and regulations as approved by the Duke University Medical Center Institutional Review Board. An example trial sequence from the Giattino et al.^48^ paradigm is illustrated in Fig. 1. Participants were asked to fixate on a central, white square for the entire trial sequence. Each trial began with a fixation screen (jittered 900–1100 ms) followed by a brief *cue array* (17 ms, one screen refresh). This cue array included two potential cue locations, each designated by four black dot masks (3.5° × 3.5° visual angle). These masked cue-locations were always symmetric across the vertical midline and randomly set across either the upper or lower visual hemifield. In a random 80% of trials, a cue (3° in diameter) would appear in one of these two cue-locations, randomly in either the left (50% of cued-trials) or right (50% of cued-trials) cue-location (*cue-present trials*). The cue was randomly either a face (50% of cue-present trials) or house (50% of cue-present trials) image. In a random 20% of trials, no cue would appear (*cue-absent trials*). The cue array also had fourteen circle distractors (3° in diameter), which were scrambled face and house images that were spatially jittered ± 0.67° in the X and Y dimensions on each trial.

On every trial, the cue array would offset at 17 ms, but the masks and fixation remained on screen (jittered 200–300 ms), a presentational pattern that enables elicitation of the OSM effect. In a random 70% of trials, a target would appear as a white square outlining one or the other of the two masks. Participants were instructed to report the location of this target as quickly and accurately as possible via a button press on a standard keyboard (100–1000 ms response window post-target). In the other 30% of trials, a target would not appear (*target-absent trials*). Lastly, participants were presented with a self-paced, three-alternative force-choice report to indicate whether a cue had been present on that trial, by reporting its left or right location or that it had been absent. Whether the cue was a face or a house stimulus was irrelevant to the participants' task.

Participants first completed a practice run of 80 trials to become accustomed to responding to the target and to making the cue report. They then completed 1000 trials of the main task, which were evenly divided into 25 equally sized experimental blocks. All stimuli were presented on a medium grey background using a 24-in LCD monitor with a screen refresh rate of 60 Hz. Participants completed all experimental procedures within a dimly lit, electronically shielded room.

### EEG data recording and preprocessing

EEG data were recorded using a 64-channel, custom-designed, extended-coverage cap^74^ with active electrodes (actiCAP, Brain Products GmbH, Gilching, Germany) and an online right-mastoid reference. Although the custom montage does not overlap perfectly with the standard 10–10 montage, each electrode location was less than  2 cm from a standard 10–10 site. Therefore, electrodes sites are reported here as those that are closest to the 10–10 montage.

The EEG data were sampled online at 500 Hz per channel using a three-stage cascade integrator-comb filter with a corner frequency of 130 Hz (actiCHamp, Brain Vision LLC, Cary, NC, USA). Two horizontal electrooculogram (EOG) channels lateral to the outer canthus of each eye and one vertical EOG channel below the left eye were used to monitor for horizonal eye movements and blinks, respectively, and for later eye artifact removal.

Data were preprocessed offline using a combination of functions from the Fieldtrip^75^ and ERPLAB^76^ toolboxes. First, data were filtered offline with a low-pass filter of 60 Hz, down sampled to 250 Hz, and then filtered again with a high-pass filter of 0.1 Hz. Next, data were re-referenced to the algebraic average of the left and right mastoids, epoched into 3-s epochs from -1000 to 2000 ms relative to cue onset, and baselined corrected from -200 to 0 ms. Trials with eye blinks or eye movements that occurred around the cue presentation were removed from all analyses using the *pop_artstep_EEGlab* function in EEGlab. Specifically, data were submitted to an algorithm using a 200 ms wide window moving across the epoch from − 100 to 500 ms in 50 ms steps, and epochs with peak-to-peak voltage differences exceeding 50 μV (for blinks) or 20 μV (for horizontal eye movements) in the corresponding EOG channels were marked for rejection. Independent component analysis (ICA) was used to correct for eye-related artifacts that occurred across the rest of the epoch, with no more than three ICA components removed for such artifacts. Trials were also excluded if they contained high-amplitude noise or excessive muscle activity (> ± 75 μV). Excessively noisy channels were interpolated using a spherical spline procedure^77^.

Frequency decomposition was performed on the epoched, preprocessed data using the *mtmconvol* method within the *ft_freqanalysis* function of Fieldtrip. This method constructs the wavelet by time-point wise multiplying the real cosine and imaginary sine component at each frequency with a specified taper. The data and tapered wavelet are then Fourier-transformed and element-wise multiplied in the frequency domain, and then the inverse Fourier transformation is computed. In the current work, this frequency decomposition was performed for each trial on frequencies between 1 and 60 Hz from -1000 to 2000 ms around the cue stimulus, in moving-window steps of 50 ms. A Hanning taper was used with window widths of 3 cycles for ≤ 7 Hz, 5 cycles for 8–14 Hz, 7 cycles for 15–30 Hz, and 10 cycles for 31–60 Hz, giving a window width for the alpha band of ~ 500 ms, shifted in 50 ms steps. Data were then log transformed with a natural base. No baseline correction in the frequency domain was performed.

### Data analyses

#### Reports of cue perception

The rates of cue-perceived and not-perceived trials were measured as a percentage of all cue-present trials. To test for possible differences in cue-perception between the face- and house-cue stimuli, we used a trial-level Bayesian mixed-effect regression analysis that modeled *cue-perception* (0 or 1) with *cue type* (house [reference] or face) as a fixed effect and with random intercepts and slopes for cue type for each participant. A standard normal prior was used over the fixed-effect coefficient, with a mean of 0 and a standard deviation of 1. A Student *t*-distribution prior was used over the intercept, with a mean of 0, a standard deviation of 2.5, and 3 degrees of freedom. For the fixed-effect coefficient (*b*), we reported the posterior mode (i.e., the “peak” of the posterior distribution), the 95% High Density Interval (HDI; reflecting the smallest credible interval that contained 95% of the values), and the BF, with BF_10_ > 10 interpreted as evidence favoring the alternative hypothesis.

#### Examining prestimulus alpha activity and reported cue perception

Averaged prestimulus alpha (8–12 Hz) power was computed for cue-perceived and not-perceived trials. For this purpose, a spatial ROI was selected that consisted of occipital and parietal scalp electrode sites (PO_z_, PO_3_, PO_4_, PO_7_, PO_8_ O_z_, O_1_, O_2,_ P_3_, P_4_). These electrode sites were selected based on past research showing that prestimulus alpha power effects on conscious perception are typically observed within occipital and/or parietal scalp locations, although there has been considerable variability in which electrode sites are analyzed (e.g.,^21,23,25,27,29^). Prestimulus alpha effects have also been observed within occipitoparietal regions even when electrode sites across the entire scalp were analyzed (e.g.,^31–33^).

For each participant, and separately for cue-perceived and not-perceived trials, power was averaged across 8–12 Hz and across the occipitoparietal spatial ROI at -250 ms pre-cue. We selected -250 ms because it was the midpoint of the last moving 500-ms time window before the cue onset that contained prestimulus activity only.

Using the *brms* package in R^78–81^, a Bayesian generalized mixed-effect regression was used to model averaged *prestimulus alpha power* with *perception* (not-perceived [reference level] or cue-perceived) as a fixed-effect and with random intercepts for each *participant.* A standard normal prior was used over the fixed-effect coefficient, with a mean of 0 and a standard deviation of 1. A Student *t*-distribution prior was used over the intercept, with a mean of 0, a standard deviation of 2.5, and 3 degrees of freedom. Again, we reported the posterior mode (*b*), the 95% HDI, and the BF, with BF_10_ > 10 interpreted as evidence favoring the alternative hypothesis.

Prestimulus alpha phase was measured at 10 Hz at -250 ms at electrode POz, a relatively straightforward approach that was similarly done in past research^27^. This approach ensured that phase measures were not obfuscated by averaging over electrodes that could reflect a mix of somewhat different cortical sources with slightly different timing or desynchronized phases. Then the preferred prestimulus alpha phase was computed separately for cue-perceived and not-perceived trials using a bootstrap procedure to match the number of trials for each condition. For this bootstrap procedure, for each subject and separately for cue-perceived and not perceived trials, a random sample of 50 trials were selected with replacement. The alpha phase was extracted for each of the 50 selected trials. The preferred prestimulus alpha phase was then computed across the sample. This process was then iterated 10,000 times and the preferred prestimulus alpha phase for all 10,000 samples was computed as the preferred prestimulus alpha phase for that subject. This resulted in one preferred prestimulus alpha phase measure for cue-perceived trials and one measure for not-perceived trial for each subject. A Bayesian generalized mixed-effect regression analysis was used to model preferred phase (in radians) with *perception* (not-perceived [reference level] or cue-perceived) as a fixed-effect and with random intercepts for each *participant.* A von Mises prior (bound by − π, π) was used with mean of 0 and a standard deviation of 1. Again, we reported the posterior mode (*b*), the 95% HDI, and the BF, with BF_10_ > 10 interpreted as evidence favoring the alternative hypothesis.

#### Examining how the VAN-window ERP varied with prestimulus alpha power

To examine the relationship between prestimulus alpha power and VAN-window ERP activity, trials were organized into terciles according to ascending prestimulus alpha power as averaged within the previously described ROI. Thus, three categorical conditions were created, with the *low-power* condition containing the tercile of trials with the lowest prestimulus alpha power, and the *high-power* condition containing the tercile of trails with the greatest power. Cue-evoked ERPs were selectively averaged within each prestimulus alpha tercile condition, time-locked to the onset of the cue. For each participant, and separately for the low- and high-power conditions, ERP amplitudes were averaged within 150–250 ms across the same occipitoparietal ROI as used to measure prestimulus alpha power. We selected this 100 ms time window to measure the VAN-window ERP because 100 ms is about the same period as one alpha cycle, and thus any differential alpha overlap would be averaged out^82^.

To simplify our statistical approach, we only compared averaged ERP amplitudes between the low-power and high-power conditions. Specifically, *VAN-window ERP amplitudes* were modeled with a Bayesian generalized mixed effect regression analysis with *alpha condition* (low-power or high-power) as a fixed-effect and with random intercepts for each *participant*. A standard normal prior was used over the fixed-effect coefficients, with a mean of 0 and a standard deviation of 1. A Student *t*-distribution prior was used over the intercepts, with a mean of 0, a standard deviation of 2.5, and 3 degrees of freedom. We reported the posterior mode (*b*), the 95% HDI, and the BF, with BF_10_ > 10 interpreted as evidence favoring the alternative hypothesis.

The rate of cue perception between the low- and high-power conditions was compared using a Bayesian repeated measures ANOVA with default priors^83,84^. A BF_10_ > 10 interpreted was considered evidence favoring the alternative hypothesis.

The topographic distributions of the ERP difference waves between the low- and high-power conditions were derived. Then the averaged difference from 150 to 250 ms post-cue was computed separately for trials when the cue appeared in the lower visual field (cue-down) and those when it appeared in the upper visual field (cue-up). Then measures of this averaged difference was extracted from three medial electrode sites (POz, CPz, FCz). These difference measures were modeled using a Bayesian generalized mixed-effect regression as a *cue location* (cue-down [reference level] or cue-up) by *electrode site* (POz [reference level], CPz, FCz) interaction with random intercepts for each *participant*. A standard normal prior was used over the fixed-effect coefficients, with a mean of 0 and a standard deviation of 1. A Student *t*-distribution prior was used over the intercepts, with a mean of 0, a standard deviation of 2.5, and 3 degrees of freedom. We reported the posterior mode (*b*), the 95% HDI, and the BF, with BF_10_ > 10 interpreted as evidence favoring the alternative hypothesis.

#### Prestimulus alpha activity on VAN-window ERP toward conscious perception

A trial-level Bayesian structural equation model with non-linear smooth terms was used to concurrently investigate (1) the effects of prestimulus alpha power and phase on stimulus-evoked VAN-window ERP amplitudes, and (2) the effects of prestimulus alpha activity (power and phase) and VAN-window ERP amplitude on cue perception. This approach offered several advantages. A non-linear regression was necessary for investigating effects of prestimulus alpha phase. Moreover, we had predicted that both the power and phase of the prestimulus alpha could impact stimulus-evoked VAN-window ERP activity and that this would have a cascading impact on cue perception. However, it was possible that prestimulus alpha power and phase would additionally have direct effects on cue perception. Thus, the structural equation model allowed us to test these complex and multifactorial relationships within one culminating model.

For each trial, we calculated a single measure of prestimulus alpha power, prestimulus alpha phase, and VAN-window ERP amplitude for each trial. Specifically, prestimulus alpha power was averaged at -250 ms across 8–12 Hz and across the occipitoparietal spatial ROI. Prestimulus alpha phase was measured at -250 ms at posterior electrode POz. VAN-window ERP amplitude was measured averaged across 150–250 ms and across the same posterior ROI as prestimulus alpha power. Prestimulus alpha power and VAN-window ERP amplitudes were then *z*-scored standardized for each participant, so that participant-level means were zero-centered with a standard deviation of 1.

We then modeled trial-level *VAN-window ERP amplitude* as a *prestimulus alpha power* by *prestimulus alpha phase* two-way non-linear interaction. Cue perception (not-perceived or perceived) was modeled as a *prestimulus alpha power* by *prestimulus alpha phase* by *VAN-window ERP amplitude* three-way non-linear interaction with random intercepts for each *participant*. A standard normal prior was used over the fixed-effect coefficients, with a mean of 0 and a standard deviation of 1. A Student *t*-distribution prior was used over the intercepts and the smooth terms, with a mean of 0, a standard deviation of 2.5, and 3 degrees of freedom.

For each coefficient (*b*), we report the maximum posterior median for positive effects and the minimum posterior median for negative effects, the 95% HDI, and the BF, with BF_10_ > 10 interpreted as evidence favoring the alternative hypothesis. Simple effects for each predictor variable are reported holding all other predictor variables at 0 (i.e., the mean). Interactions are reported as the point with the largest slope between the predictor variables.

## Data Availability

All deidentified data and code that support the findings of this study are available from the corresponding author upon request.
